# Cabrol operation in prosthetic valve infective endocarditis

**DOI:** 10.1186/1749-8090-8-S1-P18

**Published:** 2013-09-11

**Authors:** M Hıdıroglu, A Kucuker, L Cetin, A Kunt, KE Erdogan, E Sener

**Affiliations:** 1 Cardiovascular Surgery, Ataturk Training and Research Hospital, Ankara, Turkey; 2 Cardiovascular Surgery, Konya Training and Research Hospital, Konya, Turkey

## Background

Surgical therapy of prosthetic valve endocarditis is a challenging issue with different surgical strategies. We present a patient with early endocarditis treated with aortic bioprosthesis with Cabrol operation.

## Methods

A 50 years old women who had underwent aortic valve replacement (19#HPsjm) + mitral valve replacement (29#sjm) and tricuspid DeVega anuloplasty at our department, was admitted to our hospital with fever. Echocardiography confirmed vegetations on aortic and mitral valves, abscess formation in aortic root, grade IV SEK in left atrium with pulmonary artery pressure of 60-65 mmHg. With positive blood culture, vankomicin + gentamicin medication was ordered. On the follow up, vegetations on the mitral valve disappeared and the ones on aortic cusps regressed in contrary to aortic root abscess. After obtaining negative blood cultures, she was scheduled for an elective operation.

## Results

Subsequent to femoral cannulation, sternotomy was made. The abscess cavity was under the location of non-coronary cusp at the aortic valve prothesis and was aspirated and detached entirely. Mechanical aortic valve was resected. Coronary ostia were prepared as buttons but could not be mobilized because of tight adhesions. 21# Medtronic Freestyle-stentless aortic bioprosthesis was replaced after a 6 mm ringed PTFE graft was anastomosed to left main artery in end-to-end fashion. Middle part of the graft was incised and anastomosed to the distal part of the bioprothesis in side-to-side fashion. Other end of PTFE graft was anastomosed to right coronary ostium in end-to-end fashion. Inotropic support and IABP was required during weaning. At the intensive care unit, antibacterial theraphy was continued. The patient was discharged at the postoperative 16th day. Follow-up at two years is uneventful.

## Conclusion

We presented a patient we performed Cabrol operation with prosthesis valve endocarditis with aortic bioprosthesis because we failed to mobilize the coronary ostia.

